# Assessment of the Quality of Life of Children and Adolescents with Rheumatic Heart Disease in Moi Teaching and Referral Hospital Eldoret, Kenya

**DOI:** 10.3390/children13050623

**Published:** 2026-04-30

**Authors:** Myra Maghasi Koech, Njie Albertine Enjema, Juddy Wachira

**Affiliations:** 1 Department of Child Health and Paediatrics, School of Medicine, Moi University, P.O. Box 4606, Eldoret 30100, Kenya; 2 Academic Model Providing Access to Healthcare, P.O. Box 4606, Eldoret 30100, Kenya; njieenjema@gmail.com; 3 Department of Mental Health and Behavioral Sciences, School of Medicine, Moi University, P.O. Box 4606, Eldoret 30100, Kenya; wachirajuddy@gmail.com

**Keywords:** quality of life, rheumatic heart disease, children, low-resource settings

## Abstract

Background: Rheumatic heart disease (RHD) remains a significant public health problem in low- and middle-income countries. Beyond its clinical consequences, RHD adversely affects the health-related quality of life (HRQoL) of affected children and adolescents, their families, and healthcare systems. Addressing the HRQoL of children and adolescents with RHD will contribute to strengthening patient-centered care and policy development. Objective: To determine the health-related quality of life of children and adolescents with rheumatic heart disease attending follow-up at the pediatric cardiology clinic of Moi Teaching and Referral Hospital (MTRH), Kenya. Methods: This was a hospital-based cross-sectional study conducted between January and July 2024. A total of 171 children and adolescents aged 5–18 years were consecutively enrolled while attending follow-up at the pediatric cardiology clinic of MTRH. The EuroQol EQ-5D-Y and EQ-5D-L questionnaires were used to assess HRQoL across five domains: mobility, self-care, usual activities, pain/discomfort, and anxiety/depression. Overall HRQoL was evaluated using the EQ visual analog scale (EQ-VAS) and categorized as optimal (≥80%), suboptimal (70–79%), or poor (≤70%). Results: Overall HRQoL was optimal in 70.8% (n = 121) of participants, suboptimal in 8.2% (n = 14), and poor in 21.1% (n = 36). Impaired HRQoL was significantly associated with poor self-care (95% CI: 0.066–0.853; *p* = 0.028), anxiety/depression (95% CI: 0.111–0.678; *p* = 0.005), pain/discomfort (95% CI: 0.142–0.758; *p* = 0.009) and missing more than five school days (95% CI: 0.109–0.584; *p* = 0.001). Caregiver characteristics (age, education level, and income), surgical correction, RHD-related hospital admissions, comorbidities, and Ross classification were not significantly associated with HRQoL. Conclusion: Health-related quality of life among children and adolescents with RHD was most adversely affected in the mental health and mobility domains. Routine assessment of HRQoL should be incorporated into the clinical care of children and adolescents with RHD to reduce disease-related morbidity and support holistic management.

## 1. Introduction

Rheumatic heart disease (RHD) remains a significant cause of morbidity and mortality in low- and middle-income countries [1,2,3]. In East Africa, the prevalence of RHD among school-aged children and adolescents has been estimated at 17.9 cases per 1000 children, a substantially higher burden compared to that observed in high-income countries [4]. Over a six-year period, a meta-analysis reported a disease progression rate of 7.5% among children and adolescents with established RHD, rising to 11.3% among those with borderline RHD [5]. Globally, RHD has been associated with an estimated 10.5 million disability-adjusted life years (DALYs) over a 25-year period [6]. In Kenya, cardiovascular diseases contribute approximately 6.3% of total DALYs, underscoring the substantial burden of RHD within the broader spectrum of cardiovascular disease [7].

Beyond the traditional clinical focus on diagnosis and treatment of the biophysical manifestations of disease, evidence from multiple studies demonstrates that RHD significantly impairs health-related quality of life (HRQoL). Affected domains include pain, mobility, anxiety/depression, usual activities, and self-care [8,9,10,11]. Impaired HRQoL may result in school absenteeism, social exclusion, and reduced participation in age-appropriate activities, ultimately limiting a child’s ability to reach their full potential. Consequently, children with RHD may fail to meet the World Health Organization’s definition of health, which encompasses complete physical, mental, and social well-being rather than merely the absence of disease [12].

Several predictors of HRQoL among children and adolescents with RHD have been identified. In Ethiopia, Tadele et al. demonstrated that low family income and age below 20 years were associated with increased odds of poor subjective well-being, while good medication adherence and strong family psychological support were associated with improved HRQoL [11]. Additionally, surgical correction or replacement of diseased rheumatic valves in children and adolescents has been shown to result in significant improvements across multiple quality of life domains, particularly pain, anxiety, and depression [13].

Identifying predictors of poor HRQoL in children and adolescents with RHD is essential for the development of targeted interventions that promote holistic and integrated care for affected individuals and their families. Despite the high prevalence of acute rheumatic fever and rheumatic heart disease in Kenya, there is a paucity of data on the HRQoL of children and adolescents with RHD in this setting, particularly when compared with data from other regions of Africa and globally. This study therefore sought to address this gap by evaluating the HRQoL of children and adolescents with RHD at a tertiary referral hospital in Kenya and identifying key factors associated with impaired quality of life in this population.

## 2. Methods and Materials

### 2.1. Study Design and Setting

This was a cross-sectional, observational, quantitative, hospital-based study conducted among children and adolescents with rheumatic heart disease (RHD) attending follow-up at the pediatric cardiology clinic of Moi Teaching and Referral Hospital (MTRH), Eldoret, Kenya, between January and July 2024. The pediatric cardiology clinic is staffed by two pediatric cardiologists, consultant pediatricians, pediatric residents, and nurses, and attends to approximately 35 children and adolescents with RHD each month.

Moi Teaching and Referral Hospital is a tertiary referral facility located in western Kenya, serving an estimated catchment population of nearly 20 million people. It is the only hospital in the region with a dedicated pediatric cardiology clinic staffed by cardiologists and consequently receives referrals of most children requiring advanced cardiac evaluation from the surrounding counties.

### 2.2. Clinical Evaluation and Diagnosis

The diagnosis of acute rheumatic fever was made using the modified Jones criteria [14], while the echocardiographic diagnosis of rheumatic heart disease was based on the World Health Organization (WHO) criteria [15]. Echocardiographic assessments were performed by pediatric cardiologists and trained sonographers. The echocardiographic ultrasound used was ACUSON SC2000 prime cardiovascular ultrasound system by Siemens Medical Solutions USA, Inc. (Malvern, PA, USA). All echocardiographic studies were reviewed and confirmed by pediatric cardiologists, and patient records were subsequently archived. Children meeting diagnostic criteria for acute rheumatic fever and/or RHD were enrolled into the clinic and scheduled for routine follow-up every three months. Patients with clinical instability were reviewed more frequently, and inpatient admission was undertaken when clinically indicated. The child overall physical well-being at the time of study was categorized using the Ross heart failure classification system [16].

### 2.3. Study Population and Sampling

A total of 171 children and adolescents were enrolled following sample size estimation using Fisher’s formula at a 95% confidence level. A pre-study prevalence [5] estimate of 11.3% for RHD among children and adolescents, reported by Noubiap et al., was used for this calculation. Due to the limited number of children and adolescents with RHD on follow-up at the clinic, all eligible participants were consecutively recruited. Eligibility criteria included children and adolescents aged 5–18 years who had been on follow-up for at least three months. Participants who were acutely ill at the time of clinic visits were admitted for inpatient care.

### 2.4. Health-Related Quality of Life Assessment

Health-related quality of life (HRQoL) was assessed using the EuroQol EQ-5D-Y questionnaire. This instrument from the EuroQol Research Foundation (Rotterdam, The Netherlands) has demonstrated acceptable feasibility, construct validity, and reliability in sub-Saharan African settings, including Malawi and South Africa [17], supporting its use in resource-limited contexts. Its brevity and interviewer-administered format further enhance applicability in low-literacy populations.

The EQ-5D-Y questionnaire was administered as follows: the proxy version was used for children aged 5–7 years, the interviewer-administered version for children aged 8–15 years, and the EQ-5D-5L self-administered version for adolescents aged 16 years and older, in accordance with EuroQol Research Foundation guidelines. The questionnaire assesses five health dimensions: mobility, self-care, usual activities, pain/discomfort, and anxiety/depression. Each dimension is rated on three levels: no problems, some problems, or a lot of problems.

The questionnaire also includes a visual analog scale (EQ-VAS), which measures overall perceived health status on a scale ranging from 0 (worst imaginable health) to 100 (best imaginable health). In this study, EQ-VAS scores were analyzed as continuous variables and further categorized to describe overall HRQoL as optimal (≥80%), suboptimal (70–79%), or poor (≤70%). This was adopted from a study in Uganda on health-related quality of life by Ahmed et al. [13], as no country-specific pediatric value set is currently available for Kenya, utility-weighted index values were not calculated, and descriptive EQ-VAS scoring was used instead.

The EQ-5D-Y questionnaire was translated into Kiswahili by the EuroQol Group. Authorized translated copies were obtained from the EuroQol Research Foundation for use in this study (Authorization ID: 58330).

### 2.5. Assessment of Medication Adherence

Medication adherence was defined as the extent to which participants took prescribed medications as directed by healthcare providers. Adherence was assessed through self-report or proxy-report and categorized as perfect (90–100%), good (75–89%), average (50–74%), or poor (<50%), based on the proportion of missed medication days per week. To reduce recall bias, reported adherence was cross-checked against clinic records where available.

### 2.6. Ethical Considerations

Ethical approval was obtained from the Institutional Ethics and Review Committee (IREC) of Moi Teaching and Referral Hospital and Moi University (Approval No. 0004616). Administrative approval was granted by the Chief Executive Officer of MTRH. Written informed consent was obtained from parents or guardians in either English or Kiswahili, depending on language preference. Verbal assent was obtained from participating children and adolescents. Study procedures were explained in age-appropriate language, with increasing levels of detail provided to older participants to ensure comprehension.

## 3. Data Analysis

Data analysis was performed using STATA version 16 (StataCorp. 2019. Stata Statistical Software: [16]. StataCorp LLC, College Station, TX, USA). Preliminary analyses involved summarizing the demographic and clinical characteristics of the study participants. Categorical variables, including sex, education level, occupation, household size, and presence of comorbidities, were summarized using frequencies and corresponding percentages. Continuous variables such as age, caregiver income, and number of household members were summarized using means and standard deviations or medians and interquartile ranges, as appropriate.

The characteristics of rheumatic heart disease-related valve lesions were summarized as frequencies and percentages. Health-related quality of life (HRQoL) among children and adolescents with RHD was assessed using the EuroQol questionnaire, as described in Section 2, and summarized using frequencies and percentages. A 95% confidence interval was calculated for overall HRQoL.

Associations between HRQoL and categorical variables were assessed using the chi-square test or Fisher’s exact test, as appropriate. Associations between HRQoL and continuous variables were examined using analysis of variance (ANOVA) or the Kruskal–Wallis test, depending on data distribution. A stepwise ordered logistic regression model was fitted to identify participant characteristics independently associated with HRQoL. Statistical significance was defined as a *p*-value < 0.05.

## 4. Results

The results are based on 171 children and adolescents with rheumatic heart disease (RHD) who had been on follow-up for at least three months at the pediatric cardiology clinic of Moi Teaching and Referral Hospital (MTRH) by January 2024. All 171 eligible participants who were approached for enrolment provided informed consent and/or assent, and there were no refusals.

### 4.1. Sociodemographic and Clinical Characteristics

The mean age of participants at initial diagnosis was 9.2 ± 3.5 years, while the median age at the time of the study was 15 years (IQR: 12–17). Approximately three-quarters (77.7%) of caregivers had attained at least secondary-level education. Slightly more than half of caregivers (55.6%) were formally employed. Monthly caregiver income ranged from USD 7.7 to USD 462, with a median income of USD 154 (IQR: 77–231). The mean age of caregivers was 39.7 ± 7.9 years.

More than half of the participants (52.0%) lived in two-bedroom houses, followed by those living in three-bedroom houses (35.7%). The mean household size was 5.5 ± 1.9 members. Detailed sociodemographic characteristics are presented in Table 1.

### 4.2. Clinical Characteristics

The majority of participants (75.3%) had not experienced an RHD-related hospital admission in the three months preceding the study, while only seven participants (4.1%) had more than one admission. More than half of the participants (57.6%) had missed school due to RHD during this period.

Fourteen participants (8.2%) had documented comorbidities, while 25 (14.7%) had undergone surgical correction of their cardiac lesions. Eleven participants (6.5%) reported having a sibling with RHD. Regarding medical therapy, 39 participants (22.8%) were receiving triple heart failure therapy, 23 (13.5%) were on dual therapy, and 10 (5.8%) were on monotherapy. All participants (100%) were receiving monthly benzathine penicillin prophylaxis, and 25 (14.7%) were on anticoagulant therapy.

Self-reported treatment adherence was high, with 98.8% of participants reporting perfect adherence. Concurrent illnesses were reported in 8.2% of participants and included asthma, gastritis, Marfan syndrome, and malaria. Clinical characteristics are summarized in Table 2.

### 4.3. Characteristics of RHD Valve Lesions

The mitral valve was the most commonly affected valve (56.7%), followed by the aortic valve (25.7%). Mixed valve involvement involving the aortic, mitral, and pulmonary valves was observed in 8.8% of participants, while isolated tricuspid valve involvement was present in 8.8%.

Most valve lesions were classified as mild (56.7%), while 23.4% were moderate and 12.9% were severe. The predominant lesion type was regurgitation, observed in 94.2% of participants. Stenotic lesions were uncommon (5.3%), and only one participant (0.6%) had mixed regurgitant and stenotic disease. These findings are detailed in Table 3.

### 4.4. Quality of Life of Children and Adolescents with RHD

Overall health-related quality of life (HRQoL) was reported as optimal in 70.8% (n = 121) of participants, suboptimal in 8.2% (n = 14), and poor in 21.1% (n = 36).

**Figure 1:**
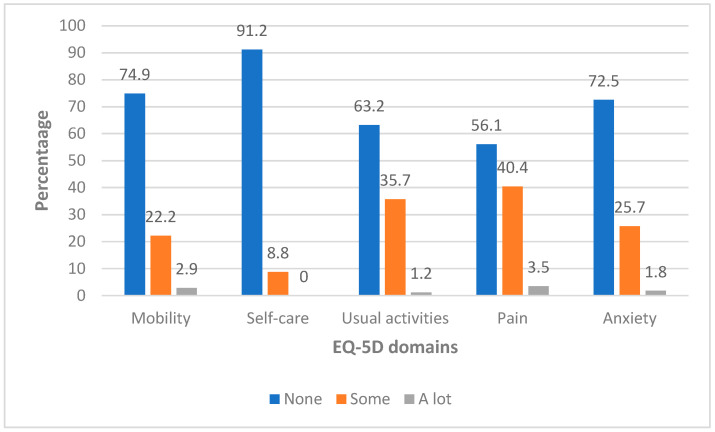
EQ-5D domain distribution.

Across the five EQ-5D domains, most participants reported no problems with self-care (91.2%) or mobility (74.9%). Additionally, 72.5% of participants reported no symptoms of anxiety or depression. However, 22.2% reported mobility limitations, 35.7% experienced difficulties with usual activities, 40.4% reported some degree of pain or discomfort, and 27.5% reported anxiety or depressive symptoms. HRQoL domain distributions are presented in Table 4 and Figure 1.

### 4.5. Factors Associated with Quality of Life

Several sociodemographic and clinical factors were significantly associated with HRQoL. House size showed a significant association (*p* = 0.005), with participants living in one-bedroom houses more likely to report poor HRQoL compared to those residing in two- or three-bedroom houses. Missing more than five school days was strongly associated with poor HRQoL, with 43.8% of these participants reporting poor HRQoL compared to 12.3% among those who missed fewer days (*p* < 0.001). Having a sibling with RHD was also significantly associated with poorer HRQoL (*p* = 0.040).

Significant associations were observed between poor HRQoL and impairments in mobility, self-care, usual activities, pain/discomfort, and anxiety/depression (all *p* < 0.001). In contrast, caregiver age, caregiver education level, caregiver income, history of surgical correction, recent RHD-related admissions, comorbidities, adjuvant therapy, and Ross heart failure classification were not significantly associated with HRQoL.

**Figure 2:**
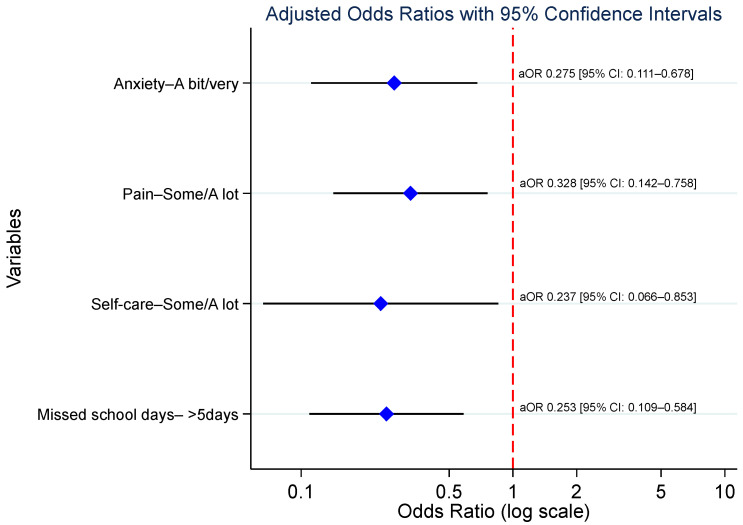
Patients’ characteristics associated with EuroQoL.

On multivariable stepwise ordered logistic regression analysis, anxiety or depression was independently associated with reduced odds of optimal HRQoL (adjusted odds ratio (aOR) = 0.275; *p* = 0.005). Experiencing pain or discomfort was associated with a 67.2% reduction in the odds of optimal HRQoL (aOR = 0.328; *p* = 0.009). Problems with self-care demonstrated a strong negative association, reducing the odds of optimal HRQoL by 76.3% (aOR = 0.237; *p* = 0.028). Missing more than five school days was associated with a 74.7% reduction in the odds of optimal HRQoL (aOR = 0.253; *p* = 0.001). These findings are summarized in Table 5 and Figure 2.

## 5. Discussion

### 5.1. Summary of Key Findings

This cross-sectional study aimed to describe the type and severity of rheumatic heart disease (RHD) valve lesions, assess health-related quality of life (HRQoL), and identify factors associated with impaired HRQoL among children and adolescents with RHD who had been on follow-up for at least three months at the Moi Teaching and Referral Hospital (MTRH) pediatric cardiology clinic.

### 5.2. Sociodemographic Characteristics

The mean age of the 171 participants at initial diagnosis was 9.2 years (SD 3.5), while the median age at the time of the study was 15 years. Slightly over half of the participants were female (50.6%). These findings are comparable to reports from Indonesia [18,19,20], Ethiopia [21], The Gambia [22], and Brazil [8], where females also predominated and the median age at diagnosis ranged between approximately 10 and 13 years.

### 5.3. Echocardiographic and Clinical Findings

In the present study, the mitral valve was the most commonly affected valve (56.1%), predominantly presenting with regurgitant lesions. The majority of participants had trivial, mild, or moderate disease severity (87.1%). Similar patterns of mitral valve predominance have been reported in studies from Ukraine [23], Africa [21,22], and Asia [20]. In a study of school-going children by Kazahura et al. in Tanzania [1], the majority of their participants were diagnosed with RHD which was primarily mitral valve disease, similar to the findings of the current study.

Regarding clinical severity, 97.1% of participants were classified as Ross heart failure class I or II, indicating mild disease. This contrasts with findings from Ethiopia, where 89.7% of participants presented with advanced heart failure (Ross class IV) [21], and from The Gambia, where nearly 57% had late-stage disease [22] This difference could be explained by differences in study setting as the current study enrolled participants on follow-up in clinic while the participants in the Ethiopian study were in-patients. Additionally, the participants in the Gambian study were invited for enrollment from their homes where the diagnosis of RHD was made, and only 32.2% had previously sought medical treatment leading to late-stage disease diagnosis. Similarly, the REMEDY sub-analysis reported severe valvular disease in approximately half of participants [24]. This difference could be explained by the fact that the remedy study reported pooled analysis of valvular disease and clinical status across 12 African countries while the current study is a single center study. Furthermore, 42.1% of participants in this study were receiving anti-heart failure medications, which may have improved clinical status. Notably, 14.6% had previously undergone mitral valve replacement surgery, suggesting that some participants initially had severe disease but were clinically stable at the time of evaluation.

Adherence to benzathine penicillin prophylaxis was nearly universal (99.4%) in this cohort, substantially higher than rates reported in Ethiopia (14.3%) [21], The Gambia (65.5%) [22], and the REMEDY study (80%) [24]. Prior studies have demonstrated that strict adherence to secondary prophylaxis reduces disease progression [25,26], which may partly explain the relatively mild disease observed in this cohort. Although clinic records were used to verify prophylaxis administration, recall bias related to self- or proxy-reported adherence could not be eliminated. Adherence in this cohort could therefore not be viewed as a standalone determinant of HRQoL. Moreover, given that almost all participants adhered (98.8%) to the prophylaxis, further stratified analysis could not be done.

### 5.4. Health-Related Quality of Life

Overall HRQoL was rated as optimal in 70.8% of participants, suboptimal in 8.2%, and poor in 21.1%. Factors significantly associated with reduced odds of optimal HRQoL included anxiety or depression (aOR = 0.275, *p* = 0.005), pain or discomfort (aOR = 0.328, *p* = 0.009), problems with self-care (aOR = 0.237, *p* = 0.028), and missing more than five school days (aOR = 0.253, *p* = 0.001). Household size was also significantly associated with poorer HRQoL (*p* = 0.005).

Compared with the Brazilian study by Carvalho et al. (2009) [8], which reported substantial impairment across physical and emotional domains using the Pediatric Quality of Life Inventory (PedsQL), the present study demonstrated greater impairment in psychological domains than in physical functioning. This difference may reflect variations in disease severity, instrument sensitivity, and sociocultural context, as the Brazilian cohort included a higher proportion of children with advanced disease. In addition, Carvalho et al. included participants with acute rheumatic fever while the current study only had participants with diagnosed RHD.

The current study demonstrates an impaired quality of life in the anxiety/depression, pain or discomfort and problems with self-care domains while other studies like Riaz and collaborates found impaired quality of life across all quality of life domains [10]. This variance could be because of different study designs as the current study is a cross-sectional study and Riaz et al. conducted a case–control study. Furthermore, Sheikh et al. found that most of their participants with RHD had anxiety/depression with a correlation between these mental health conditions and pain [3].

In India, Dixit et al. [9], using the EQ-5D-Y instrument, reported HRQoL impairments mainly driven by mobility and pain domains. In contrast, our findings indicate greater impairment in anxiety/depression and usual activities. While both the Brazilian and Indian studies identified disease severity as a key determinant of HRQoL, the present study highlights socioeconomic and psychosocial factors—such as household crowding and school absenteeism—as stronger predictors than echocardiographic severity, underscoring contextual differences in lived disease experience. Ajeng et al. in Malaysia support our findings of social economic factors such as income level and household size as pivotal determinants of quality of life in rheumatic heart disease [27]; the similarities are perhaps a result of comparable mean ages between the current study and the Malaysian cohort.

In Ethiopia, [11] using the validated Amharic WHO-5 Well-Being Index, found that low family income was associated with poorer well-being, a finding that aligns with the association between smaller household size and poorer HRQoL observed in our study. The literature from India also recognizes poor quality of life in lower- and middle-income families [9].

In Egypt, Ismael et al. [28] reported that physical, emotional, social, and school-related factors all contributed to impaired HRQoL. However, their study predominantly enrolled newly diagnosed patients, whereas the present study focused on children and adolescents who had been on follow-up for some time.

Notably, clinical disease severity, Ross heart failure classification, and prior surgical correction were not significantly associated with overall HRQoL in this cohort. Given that patients with RHD-related complications are acutely managed in the emergency and out-patient settings, which were out of the scope of the current study, these patients may not have been enrolled in this study and there were only 25 patients who had undergone surgery, hence not sufficient numbers were available for this association to be robustly analyzed. This contrasts with findings from Uganda, where Ahmed et al. [13] reported marked improvements in HRQoL following rheumatic valve replacement surgery. Dixit et al. [9] also reported post-surgical improvements in HRQoL when participants underwent balloon valvotomy and valve replacement surgery.

Overall, these findings highlight the regional relevance of psychosocial and socioeconomic determinants of HRQoL in East and sub-Saharan Africa. Unlike studies from Brazil and India that primarily linked HRQoL impairment to clinical severity, this study demonstrates that non-biomedical factors play a dominant role in shaping quality of life among Kenyan children and adolescents with RHD.

### 5.5. Study Limitations

This study has several limitations. Its cross-sectional design precludes causal inference. Reliance on self-reporting, though partially mitigated by corroborating reports with hospital records and proxy data introducing the potential for social desirability bias [29], made it difficult to further stratify the analysis in regard to its association to the HRQol. Although the EQ-5D-Y has demonstrated acceptable psychometric properties in sub-Saharan Africa, it may be less sensitive in children with chronic disease [17]. Disease-specific instruments may therefore capture additional nuances of RHD-related morbidity [30]. The single-center design also limits generalizability, although MTRH serves a diverse population, enhancing the contextual relevance of the findings.

## 6. Conclusions and Recommendations

This study demonstrates that RHD significantly impairs the quality of life of Kenyan children and adolescents, with psychosocial distress and socioeconomic factors playing a central role. These findings support a shift from a purely biomedical model of care toward a holistic biopsychosocial approach. We recommend the following:

1. Routine integration of mental health screening and pain management into RHD clinics.
2. Development of school reintegration and educational support programs to reduce school absenteeism and mitigate its impact on quality of life.

## Figures and Tables

**Table 1:** Social demographic characteristics.

Variables	Values
Child Gender	N = 171
Male	84 (49.4%)
Female	86 (50.6%)
Child age at initial diagnosis (years)	
Mean (SD)	9.2 (3.5)
Range	4–17
Child age at time of study enrollment	
Median (IQR)	15.0 (12.0–17.0)
Range	5–18
Child education level	
None	1 (0.6%)
Primary	76 (44.4%)
Secondary	86 (50.3%)
Tertiary	8 (4.7%)
Caregiver education level	
Primary	38 (22.2%)
Secondary	67 (39.2%)
Tertiary	66 (38.5%)
Caregiver’s Gender	
Male	79 (46.2%)
Female	92 (53.8%)
Caregiver’s Age (years)	
Mean (SD)	39.7 (7.9)
Range	23–62
Caregiver’s Occupation	
Formal employment	95 (55.6%)
Self-employed	11 (6.4%)
Casual laborer	14 (8.2%)
Farmer	35 (20.5%)
Unemployed	16 (9.4%)
Caregiver’s Income (USD)	
Median (IQR)	154 (1)
Range	7.7–462
House Size	
Single room	20 (11.7%)
One bedroom	1 (0.6%)
Two bedrooms	89 (52.0%)
Three bedrooms	61 (35.7%)
Number of Household Members	
Mean (SD)	5.5 (1.9)
Range	2–12

**Table 2:** Clinical characteristics.

Variables	Values
RHD-related admissions in the last 3 months	N = 171
0	129 (75.4%)
1	35 (20.5%)
2	6 (3.5%)
3	1 (0.6%)
Missed school days in the last 3 months	
Never	73 (42.6%)
1–5 days	50 (29.2%)
6–10 days	6 (3.5%)
>10 days	42 (24.7%)
Other concurrent illness	
No	157 (91.8%)
Yes	14 (8.2%)
Surgical correction of lesion	
No	146 (85.3%)
Yes	25 (14.7%)
Sibling with RHD	
No	160 (93.5%)
Yes	11 (6.5%)
Treatment modality (heart failure therapy: Frusemide, spironolactone, carvedilol, enalapril and digoxin)	
Monotherapy	10 (5.8%)
Dual therapy	23 (13.5%)
Triple therapy	39 (22.8%)
Additional medication	
Benzathine penicillin prophylaxis	171 (100%)
Anticoagulant (warfarin)	25 (14.7%)
Self-reported adherence to treatment	
Good	2 (1.2%)
Perfect	169 (98.8%)

**Table 3:** RHD severity and valve lesion characteristics.

Variables	Values
Affected valve	N = 171
Aortic	44 (25.7%)
Mitral	97 (56.7%)
Mixed Aortic Mitral and pulmonary	15 (8.8%)
Tricuspid	15 (8.8%)
Severity	
Trivial	12 (7.0%)
Mild	97 (56.7%)
Moderate	40 (23.4%)
Severe	22 (12.9%)
Lesion nature	
Regurgitation & stenosis	1 (0.6%)
Stenosis	9 (5.3%)
Regurgitation	161 (94.2%)

**Table 4:** Health-related quality of life (HRQol) of the participants.

Variables	Values
Mobility	N = 171
None	128 (74.9%)
Some	38 (22.2%)
A lot	5 (2.9%)
Self-care	
None	156 (91.2%)
Some	15 (8.8%)
Usual Activities	
None	108 (63.2%)
Some	61 (35.7%)
A lot	2 (1.2%)
Pain/Discomfort	
None	96 (56.1%)
Some	69 (40.4%)
A lot	6 (3.5%)
Anxiety or Depression	
None	124 (72.5%)
A bit	44 (25.7%)
Very	3 (1.8%)
Ross heart failure classification	
Class 1	110 (64.7%)
Class 2	56 (32.9%)
Class 3	5 (2.9%)
EuroQoL (overall quality of life)	
Poor (≤70%)	36 (21.1%)
Suboptimal (71–79%)	14 (8.2%)
Optimal (≥80%)	121 (70.8%)

**Table 5:** Patient characteristics associated with EuroQoL.

Variables	aOR	*p*-Value	95% CI
Anxiety or depression			
None	1		
A bit/very	0.275	0.005	0.111–0.678
Pain discomfort			
None	1		
Some/A lot	0.328	0.009	0.142–0.758
Self-care			
None	1		
Some/A lot	0.237	0.028	0.066–0.853
Missed school days			
≤5 days	1		
>5 days	0.253	0.001	0.109–0.584

## Data Availability

The original contributions presented in this study are included in the article. Further inquiries can be directed to the corresponding author(s).
